# Genome-Wide Identification of New Reference Genes for qRT-PCR Normalization under High Temperature Stress in Rice Endosperm

**DOI:** 10.1371/journal.pone.0142015

**Published:** 2015-11-10

**Authors:** Heng Xu, Jian-Dong Bao, Ji-Song Dai, Yongqing Li, Ying Zhu

**Affiliations:** 1 State Key Laboratory Breeding Base for Zhejiang Sustainable Pest and Disease Control, Zhejiang Academy of Agricultural Sciences, Hangzhou, 310021, P. R. China; 2 Fujian-Taiwan Joint Center for Ecological Control of Crop Pests, College of Life Sciences, Fujian Agriculture and Forestry University, Fuzhou, 350002, P. R. China; 3 Key Laboratory of South China Agricultural Plant Molecular Analysis and Genetic Improvement, South China Botanical Garden, Chinese Academy of Sciences, Guangzhou, 510650, P. R. China; China National Rice Research Institute, CHINA

## Abstract

qRT-PCR is one of the most popular approaches to analyze specific gene expression level, and stably expressed reference genes are essential to obtain reliable results. However, many reference genes are only stable under certain circumstances and different reference genes might be required in different experiments. High temperature is a common stress that affects rice endosperm development and it has become a hot topic recently. Although study about reference genes at different developmental stages in rice has been reported, these genes may not be suitable to study high temperature mediated responses especially in endosperm. In our quest for proper reference genes to quantify gene expression in rice endosperm under high temperature, we studied 6 candidate genes selected from the transcriptome data and 11 housekeeping genes. All genes were analyzed with qRT-PCR and the expression stability was assessed with software geNorm and NormFinder. *Fb15* and *eIF-4a* were identified as the two most stable genes in endosperm at different developmental stages, while high temperature treatment has a least effect on expression of *Fb15* and *UBQ5* in rice endosperm. Our results provide some good candidate reference genes for qRT-PCR normalization in rice endosperm under different temperatures.

## Introduction

Quantitative reverse transcription polymerase chain reaction (qRT-PCR) is an efficient, cost-effective and reproducible method for quantifying gene transcript levels in molecular biology research [1]. qRT-PCR is widely used to analyze gene expression in plant tissues at different developmental stages and/or under different biotic or abiotic stresses [2–4]. However, the accuracy of qRT-PCR is determined by a number of factors, such as mRNA quality, reverse transcription and PCR amplification efficiency. Selection of suitable reference genes for normalization is a crucial step to obtain reliable qRT-PCR results. Good reference genes should meet certain requirements: (i) proper expression level, at least not too low to be detected or too high; and (ii) stable expression during the whole experiment. The housekeeping genes have been thought to be good reference genes since they are required to maintain basic cellular function [5–7]. However, recent studies found that the expression levels of some housekeeping genes varied significantly under some particular experimental conditions [3, 8–10]. Thus, there is an urgent need to choose the proper reference genes for specific studies.

Rice is one of the most important crops which feed the world’s largest population. Rice endosperm stores most of the seed nutrients and serves as an important food resource for human. Endosperm development is closely related to seed yield, cooking quality and taste and this process is known as a complex process controlled genetically. Many key genes, especially the key enzymes involved in metabolism of storage materials in endosperm, have been identified and functionally studied in rice. On the other hand, endosperm development can also be affected by environmental factors. High temperature conditions during ripen season often result in low yield and poor quality. Recent results suggested that some of the key enzymes in starch or storage protein synthesis responsive to high temperature are regulated at transcriptional level [11]. qRT-PCR has been and will be widely used in researches on endosperm development under normal and high temperature conditions. Although some reference genes at various developmental stages in rice have been identified based on the microarray data and bioinforamtic analysis [4, 12], there are no reference genes reported particularly during rice endosperm development, especially under different temperature conditions. Some studies in rice seed didn't confirm the reference gene expression with qRT-PCR [4]. In addition, rice seed development starts from double fertilization. Two polar nuclei in the central cell of embryo sac and one sperm nucleus produce a triploid (3n) endosperm while the egg cell and another sperm cell together generate a diploid (2n) embryo. In most of the previous studies, whole seed including embyro, endosperm, pericarp and seed coat was collected as seed sample [4]. However, the gene profiles in embryo and endosperm are dramatically different [13]. Furthermore, RNA samples collected not from the accurate time points but within some periods, such as 0–2 DAP (days after pollination), 3–4 DAP, 10–20 DAP [4] might also affect the data accuracy.

In recent years, systemic transcriptome analyses, including DNA microarray and RNA-seq, were widely used in the study of seed development [11, 13] and some of them focused on endosperm development [13]. The publicly available transcriptome data from different developmental stages of endosperm provides valuable resources that can be used to select proper reference genes [11, 13]. Besides, several algorithms such as geNorm, Bestkeeper and NormFinder [8,14,15] were developed for expression analysis. The geNorm is a popular statistical algorithm which calculates the average expression stability (M values) of all candidate reference genes in the given samples. This algorithm defined M value as the average pairwise variation of each candidate reference gene with all other candidate reference genes. The most stably expressed reference genes have the lowest M values [8]. The NormFinder is another statistical algorithm which was written as a Visual Basic application for Microsoft Excel. This algorithm calculates the expression stability for all candidate reference genes based on intra- and inter-group variations. The most stably expressed reference genes have the lowest expression stability values [15].

In this study, eight genes (*EXP1*, *RPL23a*, *SKP-1b*, *eIF-5a*, *ARP*, *SAP18*, *RPL36-2*, *Fb15*) were selected as candidate reference genes based upon their coefficient of variation (CV) and expression intensity both in our RNA-seq data from rice endosperm grown at different temperatures and the open microarray data from rice endosperm at different developmental stages (http://www.plexdb.org/plex.php?database=Rice)[13]. The expression of *RPL23a* and *RPL36-2* could not be detected by qRT-PCR. Therefore other 6 genes and 11 widely used housekeeping genes (*Actin*, *eEF-1a*, *GAPDH*, *β-TUB*, *eIF-4a*, *UBQ10*, *UBC*, *UBQ5*, *18S rRNA*, *17S rRNA* and *25S rRNA*) in rice [3, 16–18] were further analyzed. qRT-PCR combined with computational analysis showed that *Fb15* and *eIF-4a* have the most stable expression level in rice endosperm development under normal temperature, while *Fb15* and *UBQ5* are the most stable genes in rice endosperm development under high temperature. These genes can be used as the reference genes in research on endosperm development and/or effects of high temperature on grain filling.

## Materials and Methods

### Plant materials

Rice plants (*Oryza sativa* sp. *Japonica*, Nipponbare) were grown under control temperature (28°C/22°C, 12-h-day/12-h-night cycle). Caryopses were tagged at the initiation of pollination. To eliminate the negative effects of high temperature on pollination and fertilization, five days after pollination rice plants were then transferred to either a 35°C/28°C or 28°C/22°C artificial climate room for high temperature or control treatment. For RNA-seq analysis, fresh rice seeds were sampled 5 d after treatment. For short-term high temperature treatment, fresh rice seeds were sampled at 0 h, 1 h, 6 h and 12 h time points. And the samples labeled as 0H, 1H, 6H, 12H for high temperature treated samples and 0N, 1N, 6N, 12N for control samples, respectively. For long-term treatment, fresh rice seeds were sampled at 2 d, 4 d, 7 d and 9 d time points. And the samples labeled as 7DAP-H, 9DAP-H, 12DAP-H, 14DAP-H for high temperature treated samples and 7DAP-N, 9DAP-N, 12DAP-N, 14DAP-N for control samples, respectively. Samples were frozen immediately in liquid nitrogen before they were stored at -80°C for RNA extraction.

### RNA extraction and complementary DNA synthesis

Total RNA was extracted from rice endosperm using RNAplant plus kit (Tiangen), and then treated with TURBO DNA-*free* ^TM^ kit (Life Technologies) according to the manufacturers’ manual. RNA quality and concentration were determined using Beckman DU730 spectrophotometric measurement (Beckman) and the integrity of RNA was monitored by 1% agarose gel electrophoresis and ethidium bromide staining. Complementary DNA (cDNA) was synthesized using GoScript^TM^ Reverse Transcription System (Promega) from 5 μg total RNA with random primers and resulting cDNA were used for qRT-PCR analysis.

### RNA sequencing

For RNA-seq, poly (A) mRNA was isolated with Micropoly (A) Purist^TM^ mRNA Purification kit (Life Technologies) from genomic-DNA-free total RNA samples. The mRNA was then fragmented using divalent cations under elevated temperatures. cDNA library were prepared with the mRNA-seq Sample Preparation kit (Illumina) according to the protocol of the manufacturer, and sequencing of the RNA-Seq libraries was performed by Shanghai Hanyu biotech Co. Ltd with Illumina HiSeq2000 Genome Analyzer. All RNA-seq data for this article have been deposited at the National Center for Biotechnology Information Sequence Read Archive (http://www.ncbi.nlm.nih.gov/sra/) under accession number SRP063555.

### Selection of candidate reference genes

Candidate reference genes were selected from the publically available microarray database from rice endosperm at different developmental stages and our RNA-sequencing data as described above. Two open microarray datasets (GSE11966 and GSE27856) from endosperm at different developmental stages were used in this study. Five tissues including embryo, endosperm, leaf, root of 7-day-old seedlings and 10-day-old seedling were analyzed in dataset GSE11966. Rice ovary and three development stages of embryo or endosperm (embryo 3, 9, 12 DAP; Endosperm 3, 9, 16 DAP) were analyzed in dataset GSE27856. The microarray data were obtained from the Plant Expression Database. Eight genes were identified as candidate reference genes for qPCR validation based upon their CV, mean value and the number of isoforms. Gene ID of these genes is shown in Table 1.

**Table 1:** List of 8 candidate genes with proper expression intensity.

Gene name	Gene ID	Description	Primer sequences for qRT-PCR
*EXP1*	LOC_Os04g31910	Expressed protein	F:TGTGCTCAGGAGATGGGATC
			R:CACACTGAGCAAGGACTCTG
*RPL23a*	LOC_Os01g24690	60S ribosomal protein L23A	F:TCACAGACCAAAGACCCTGA
			R:TCGACGATGAAGACAAGGGT
*SKP-1b*	LOC_Os11g26910	E3 ubiquitin ligase SCF complex, Skp subunit	F: TGATCACCCTGAAGAGCTCC
			R: GCAGTACTCGATGACCTTGG
*eIF-5a*	LOC_Os07g02210	eukaryotic translation initiation factor 5A	F: GAGCGGCAACACTAAGGATG
			R: AATCTCCTTGACGGCACAGA
*ARP*	LOC_Os05g37330	60S acidic ribosomal protein	F: CTCTCAGAACTGGAGGGCAA
			R:CTTTGCCTCTTCAGCAGGTG
*SAP18*	LOC_Os02g02960	histone deacetylase complex subunit SAP18	F:GGAGCCGAAAGATGAAGTGC
			R:ACCCACCTCTTTGACAACGA
*RPL36-2*	LOC_Os05g38520	60S ribosomal protein L36-2	F:ATGGCGCCGTCGCAGCCGAAGTC
			R:GTGAGCGTGACCGCCACCAGACC
*Fb15*	LOC_Os02g07910	fiber protein Fb15	F:GAGCGGCAACACTAAGGATG
			R:AATCTCCTTGACGGCACAGA

### Primer design and real time PCR

To isolate the most stable and reliable reference genes in the process of endosperm development under different temperature conditions, specific primers for the above 8 candidate reference genes were designed using the Primer 3 (http://frodo.wi.mit.edu/primer3/). The specific primers of housekeeping genes, *Actin eEF-1a*, *GAPDH*, *β-TUB*, *eIF-4a*, *UBQ10*, *UBC*, *UBQ5*, *17S rRNA*, *18S rRNA* and *25S rRNA* were amplified as described previously [3].

To check specificity of these primers, qPCR was performed using the iQ™ SYBR® Green PCR Super Mix (Bio-Rad) on CFX96 Real-Time PCR Detection System and the PCR products were analyzed by melting curves and also on 2% ethidium bromide stained agarose gel. Each qRT-PCR reaction mix containing 0.5 μl of 1: 5 diluted cDNA, 4 μl DNase/RNase free water H_2_O, 5 μl 2× iQ™ SYBR® Green PCR Super Mix, and 0.5 μl of the forward and reverse primers. The following amplification program was used: initial denaturation at 95°C for 3 min, followed by 40 cycles of denaturation for 10 s at 95°C, annealing for 10 s at 58°C, and extension for 10 s at 72°C. Melting curve data were collected from 65°C to 95°C in 0.5°C increments. The real-time PCRs were performed on CFX96 Real-Time PCR System (Bio-Rad), the threshold Cycle (Ct) was auto-calculated by the CFX96 Manager Software (Bio-Rad).

### Gene expression stability analysis

Two widely used algorithms, NormFinder and geNorm were used to analyze the stability of the candidate reference genes under different experimental conditions. Ct values of the candidate reference genes were imported into software according to the corresponding manuals of NormFinder [15] and geNorm v3.5 [8] algorithms, respectively, to evaluate gene expression stability.

The expression stability value (M) for all genes and mean pairwise variation of a gene from all other reference genes in a given set of samples were calculated by the geNorm algorithm. All the candidate reference genes are ranked based on their stability in a given set of samples, and the number of reference genes necessary for an optimal normalization is indicated as well.

The gene expression stability with optimal normalization among a set of candidate reference genes were analyzed by NormFinder algorithm. The NormFinder ranks the stability of candidate reference genes and calculates not only the candidate reference gene variation but also the variation between sample subgroups of the sample set. The lowest stability value indicates the most stable candidate reference genes within the gene set examined.

## Results

### Transcriptome analysis of rice endosperm under high temperature based on RNA sequencing

To analyze expression profile changes caused by high temperature treatment in endosperm, rice plants Nipponbare (*Oryza sativa* sp. *Japonica*) were transferred to high temperature (35°C/28°C) or kept at control temperature (28°C/22°C) at 5 DAP. Since we focused on the durable effects of high temperature on endosperm development, RNA samples were collected 5 days later for RNA-seq. The expression level for each transcript was calculated as RPKM (Reads Per Kilobase exon Model per Million mapped reads)-derived read counts according to the number of uniquely mapped reads that overlapped with exonic regions [19]. A lower coefficient of variation (CV) indicates less variation, 712 genes with lowest CV (CV<0.2) value were selected as the genes with most stable expression under high temperature according to our RNA-seq data. All these genes were assigned to various Gene Ontology (GO) terms and they are enriched in the categories cellular process, cellular component, metabolic process, plastid, membrane and biosynthetic process.

### Selection of candidate reference genes

To further isolate the new candidate reference genes in endosperm under high temperature, we analyzed our RNA-seq data (GSE73220) and two open microarray data (GSE11966 and GSE27856) from endosperm at different developmental stages (at 3, 6, 9 and 16 DAP) [13]. Firstly, RNA-seq data and two open microarray data were used to calculate the mean expression value (MV), standard deviation (SD) and coefficient of variation (CV, CV = SD/mean) for each dataset. We used CV<0.2 as a cut-off value in selection process, and 37 genes were selected as candidate reference genes according to their CV both in RNA-seq data and two open microarray data sets (S1 Table). These genes encode proteins involved in various biological processes, such as protein synthesis and turn over (ribosomal protein and F-box proteins) and signal transduction (transcription factor). Genes involved in protein translation are also overrepresented in the list.

Besides the stability of gene expression, the expression intensity of reference genes is also important. Neither genes with high expression abundance nor lowly expressed genes are suitable for being the reference genes. *Actin* is the most commonly used reference gene in plant. Therefore it was used as the reference to select the candidate gene with proper expression intensity according to the mean expression value of two open microarray data and our RNA-seq data. Furthermore, all candidate genes with more than two isoforms gene were excluded according to alternative splicing in our RNA-seq data. Finally, eight genes that showed stable expression levels were selected as candidate reference genes according to their CV, expression intensity, and the number of isoforms (Table 1). Since the expression of *RPL23a* and *RPL36-2* could not be detected by qRT-PCR, other 6 genes were used for further analysis.

### Stability of candidate reference gene expression under high temperature in rice endosperm

**Fig 1:**
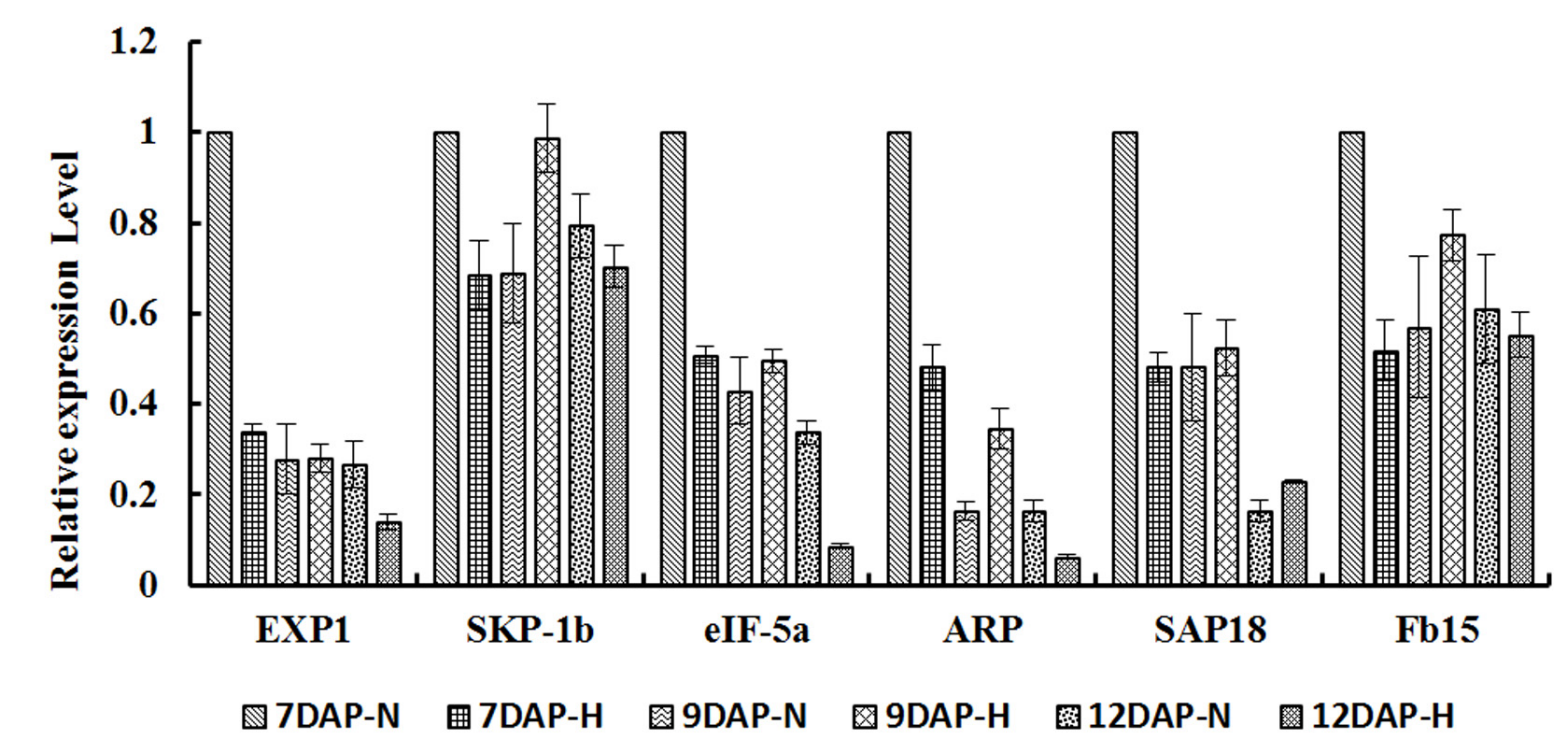
Relative expression of candidate reference genes under high temperature. Error bars show the standard deviations. All genes were compared with *18S rRNA*. The experiment was repeated three times and similar results were obtained.

Previous results indicated *18S rRNA* is more abundant and more stable than messager RNAs (mRNAs) under most of the conditions [20]. Therefore, we used *18S rRNA* as qRT-PCR reference gene to normalize the expression levels of these 6 candidate genes in rice endosperm under high temperature. We found that at different time points (2 d, 4 d, 7 d high temperature treatment, samples labeled as 7DAP-H, 9DAP-H, 12DAP-H, see Materials and Methods), two genes (LOC_Os11g26910 and LOC_Os02g07910) showed more stable expression than other 4 genes (Fig 1). LOC_Os11g26910 encodes SKP1-like protein 1B (SKP-1b), involving component in E3 ubiquitin ligase SCF complex, and LOC_Os02g07910 encodes a fiber protein Fb15. *SKP-1b* and *Fb15* both expressed at relatively stable levels in various tissues as shown in the open microarray database (https://genevestigator.com/gv/index.jsp).

### Expression intensity of the candidate reference genes

**Fig 2:**
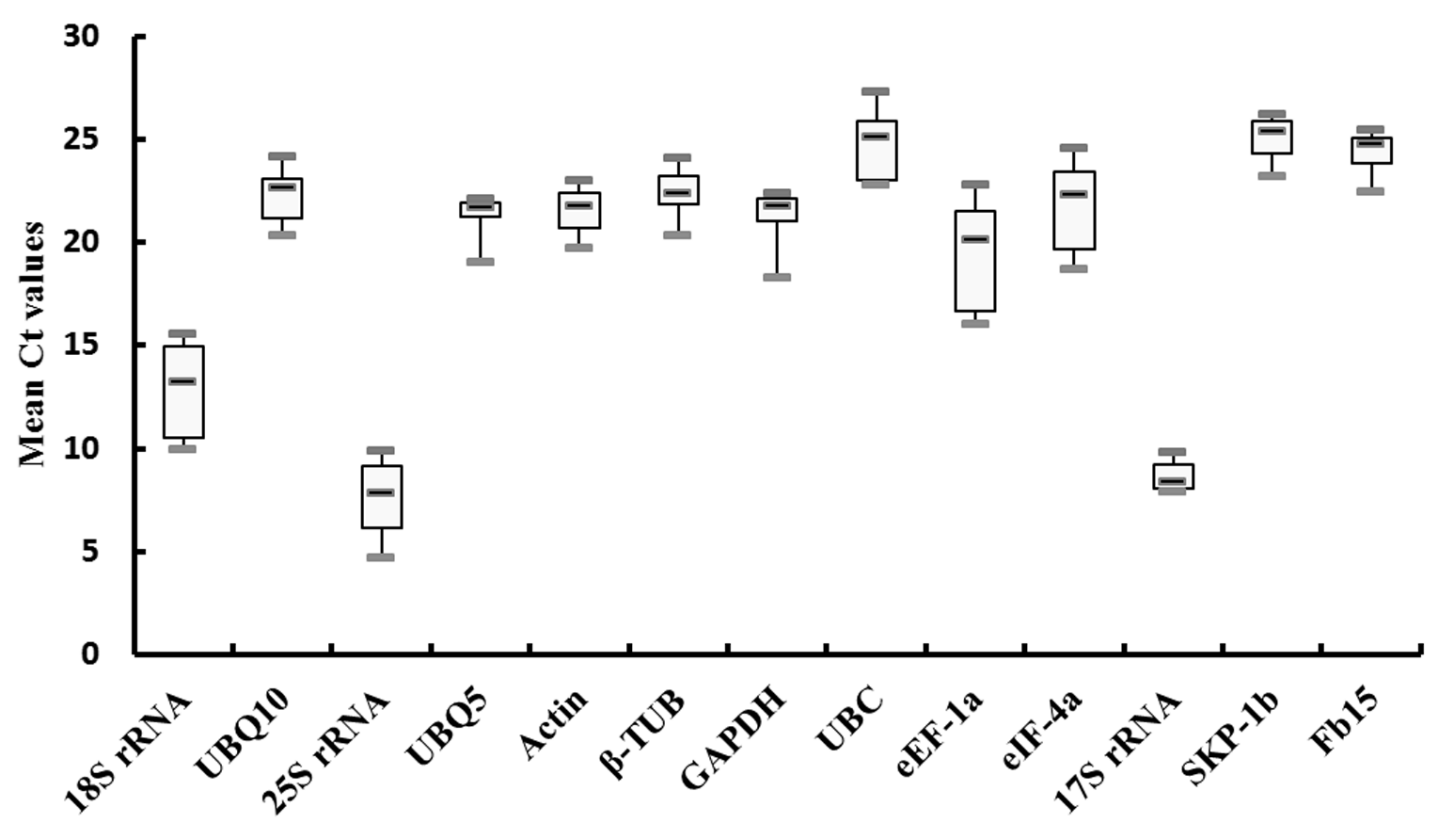
CT values of the candidate reference genes in qRT-PCR assay. Expression data displayed as CT values for each candidate reference gene in all samples. A line across the box is depicted as the median. The boxes indicate the 25th and 75th percentiles. Whiskers represent the maximum and minimum values.

To confirm these 2 candidate genes (*Fb15* and *SKP-1b*) have the proper expression intensity, their expression levels together with that of 11 widely used housekeeping genes (*Actin*, *eEF-1a*, *GAPDH*, *β-TUB*, *eIF-4a*, *UBQ10*, *UBC*, *UBQ5*, *18S rRNA*, *17S rRNA* and *25S rRNA*) were examined by qRT-PCR in all 15 samples (0N, 1N, 6N, 12N, 1H, 6H, 12H, 7DAP-N, 9DAP-N, 12DAP-N, 14DAP-N, 7DAP-H, 9DAP-H, 12DAP-H, 14DAP-H, see Materials and Methods). The expression intensity was determined by Threshold Cycle (Ct) value [21]. The mean Ct values of these genes ranged from 7.59 to 25.09 (Fig 2). Ribosome RNA genes, *25S rRNA*, *17S rRNA* and *18S rRNA*, were highly expressed and the mean values of Ct were 7.82, 8.36 and 13.19, respectively. The mean value of Ct from 10 coding protein genes was 22.31. As a result, the expression intensity of rRNA genes was about 850-folds more than that of protein coding genes. Furthermore, the mean Ct values of two candidate genes, *Fb15* and *SKP-1b*, were 24.35 and 25.09, respectively. The mean Ct values of *Fb15* and *SKP-1b* were similar to housekeeping genes, such as *ACT*, *eEF-1a*, *GAPDH*, *UBQ5*, *eIF-4a*. These results suggested that *Fb15* and *SKP-1b* have the proper expression intensity for being the reference genes.

### GeNorm analysis

To further compare the expression stability of 2 candidate reference genes (*SKP-1b* and *Fb15*) with 11 mostly used reference genes in endosperm under high temperature treatments, the geNorm algorithm was used. The geNorm algorithm calculate the average expression stability (M values) for all the reference genes [8]. The most stably expressed reference gene has the lowest M values while the least stably expressed reference gene has the highest M values. The geNorm suggests M value 1.5 as a cut off value which means that genes M value<1.5 can be used as candidate reference genes. All 13 genes were ranked according to M value in both short-term and long-term high temperature treatments (see Materials and Methods) with all below 1.5 in both treatments, indicating stable expression levels under high temperature conditions.

**Fig 3:**
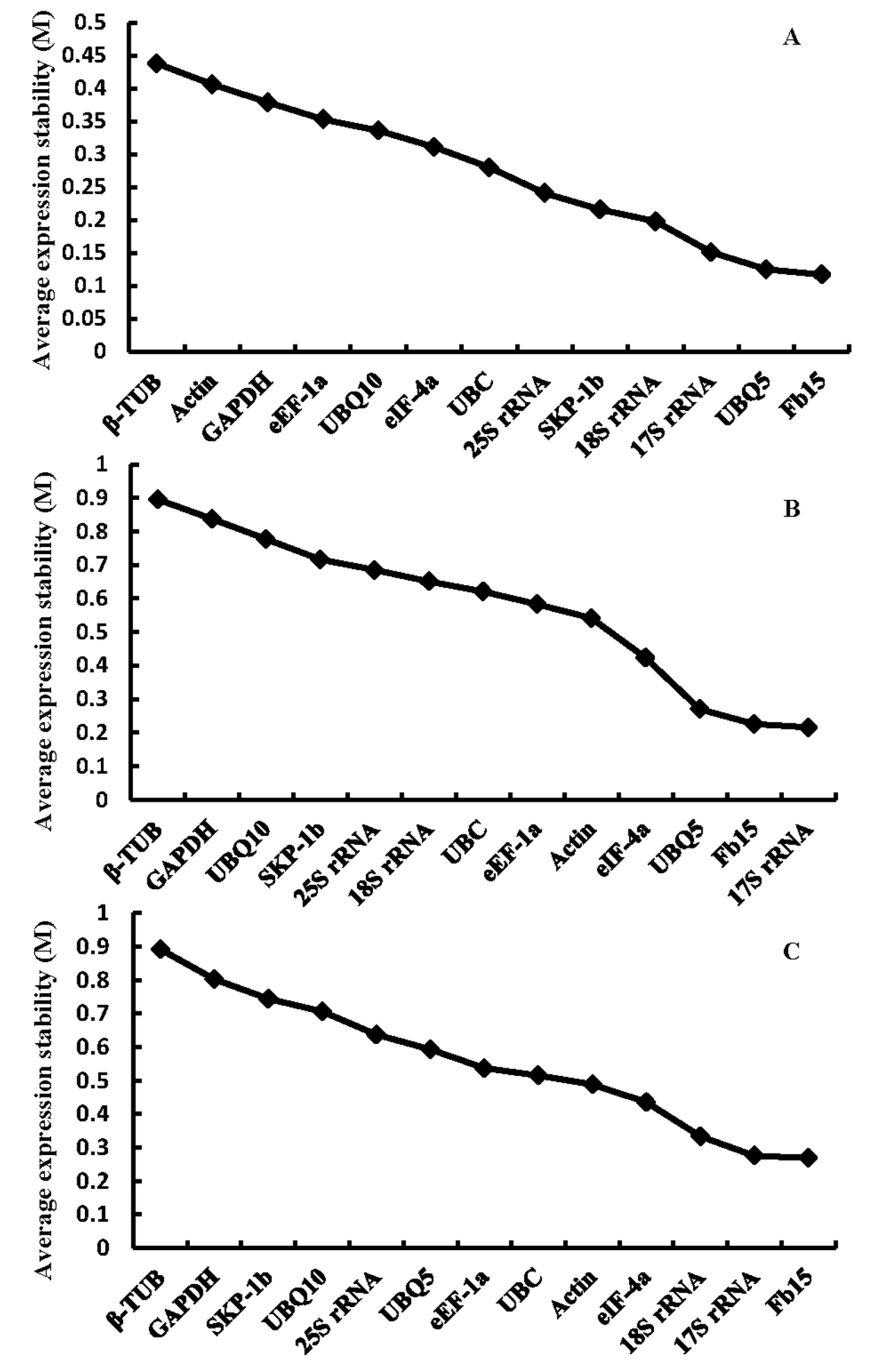
Expression stability values (M) and ranking of candidate reference genes as calculated by geNorm. Average expression stability values (M) of reference genes were measured during stepwise exclusion of the least stable reference genes under different conditions. (A) short-term high temperature treatment, (B) long-term high temperature treatment, (C) endosperm development stages.

Under short-term high temperature, *Fb15* showed the lowest M value 0.117. *UBQ5* took the second place with the M value 0.125. *β-TUB* got the highest M value 0.895 (Fig 3A). Under long-term high temperature, *17S rRNA* showed the lowest M value 0.216 while the *Fb15* took the second with M value 0.226. The difference of M values between *17S rRNA* and *Fb15* is very small at long-term high temperature (Fig 3B). As shown in Fig 3A and 3B, under both high temperature treatments, *Fb15*, *17S rRNA* and *UBQ5* were the top 3 genes with the lowest M values, i.e. the most stable expression among the 13 genes at high temperature conditions.

We further analyzed the expression stability of these 13 genes at different developmental endosperms under normal growth condition. As shown in Fig 3C, again *Fb15* stood out as the most stable gene with lowest M value while *β-TUB* showed the highest M value. These data suggested that *Fb15* is a better reference gene than the commonly used ones, such as *Actin*, *UBQ10* or *β-TUB*, in the study of endosperm development.

Furthermore, for some studies, single reference gene is inadequate and two or more stable reference genes may be required for normalization. Therefore, pairwise variation (V_n/n+1_) was calculated with geNorm to determine the optimal number of reference genes for normalization. For the most stable control genes (with lowest M value), the normalization factors (NF) were calculated, and for the rest, NFs were calculated by stepwise inclusion of one reference gene that remains most stable. V_n/n+1_ was calculated between sequential normalization factors (NF_n_ and NF_n+1_, n≥2) in stepwise manner and reflects the effect of including additional (n + 1) gene. Vandesompele (2002) proposed 0.15 as pairwise variation cut-off value and additional reference genes are not necessary if V_n/n+1_is below this value [8].

**Fig 4:**
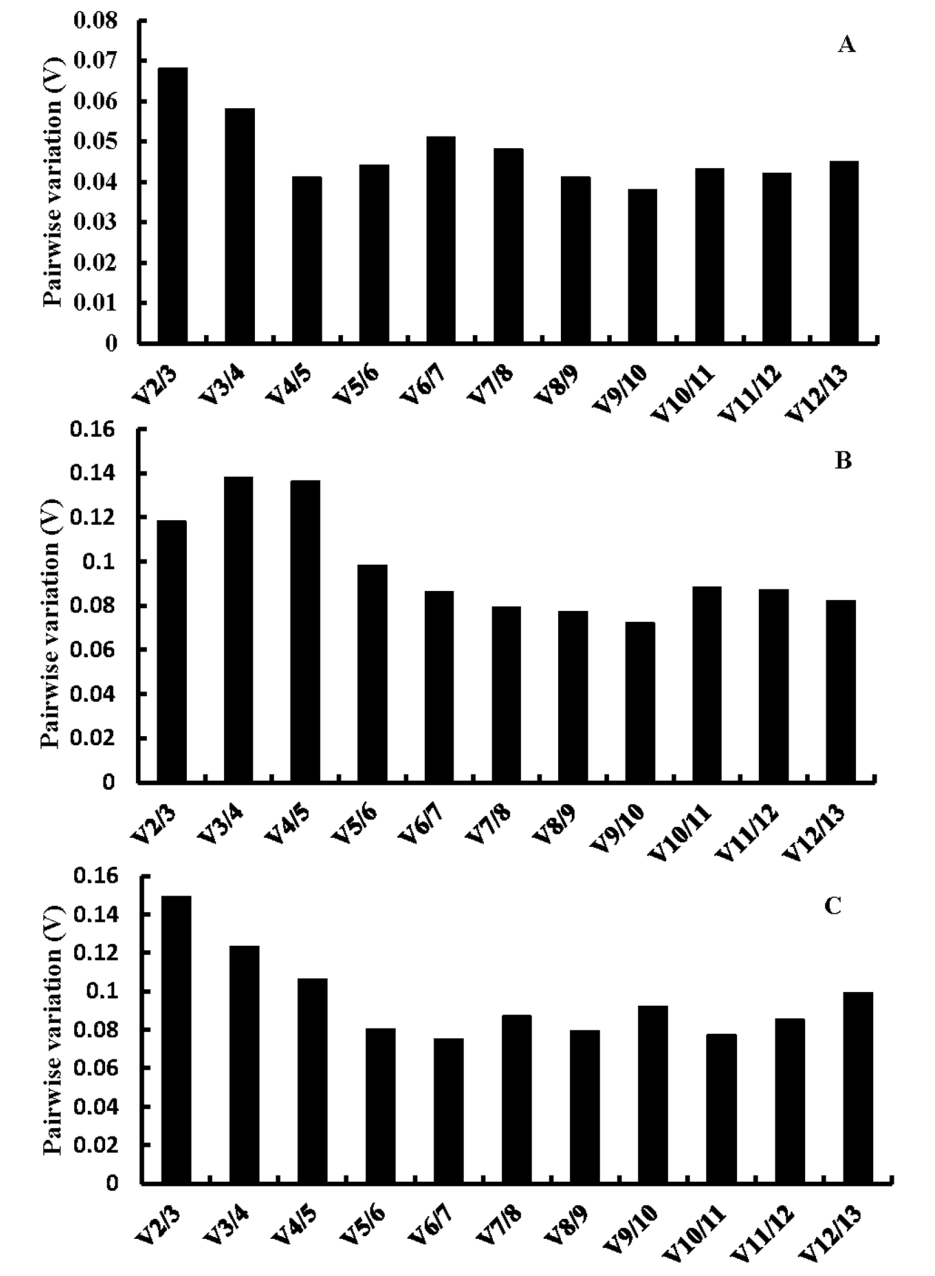
Determination of the optimal number of reference genes. Pairwise variation calculated by geNorm to determine the optimal number of reference genes for accurate normalization under different conditions. (A) short-term high temperature treatment, (B) long-term high temperature treatment, (C) endosperm development stages.

As shown in Fig 4, under all experimental conditions in this research, the V_2/3_ was below 0.15, so a third reference gene has no significant effect on pairwise variation (V_n/n+1_). Therefore, two reference genes would be sufficient for gene normalization at different developmental stages of endosperm under both normal and high temperature conditions. However, using two or more reference genes is always recommended and it can greatly improve the accuracy and reliability of the qPCR results compared to using a single reference gene.

### NormFinder analysis

To verify the results obtained from geNorm analysis, another software NormFinder was used in this study. NormFinder use a mathematical model-based approach to identify reference genes. All candidates reference gene can be ranked based on intra- and inter-group variations. Both results would be combined into a stability value for each candidate reference gene [15]. The rank of the genes from NormFinder analysis were little different with those from geNorm (Table 2), which may be due to the different statistical algorithms applied in both applications. NormFinder analysis revealed that *eIF-4a*, *17S rRNA*, *UBC* and *Fb15* were the best 4 reference genes in rice endosperm development under normal temperature. *17S rRNA*, *18S rRNA*, *UBQ5* and *Fb15* showed the most stable expression among the 13 genes under short-term high temperature treatment, while *UBQ5*, *Fb15*, *17S rRNA* and *eIF-4a* genes seem more stable under long-term high temperature treatment. We can conclude that *Fb15*, *17S rRNA* and *UBQ5* are the best 3 reference genes under both short-term and long-term high temperature stresses. These results are consistent with the one obtained from geNorm analysis.

**Table 2:** Expression stability values (M) and ranking of candidate reference genes as calculated by NormFinder.

Rank	endosperm development		short-term high temperature		long-term high temperature	
	gene	stability	gene	stability	gene	stability
1	*eIF-4a*	0.146	*17S rRNA*	0.124	*UBQ5*	0.338
2	*17S rRNA*	0.328	*18S rRNA*	0.183	*Fb15*	0.423
3	*UBC*	0.376	*UBQ5*	0.193	*17S rRNA*	0.456
4	*Fb15*	0.387	*Fb15*	0.211	*eIF-4a*	0.468
5	*18S rRNA*	0.423	*SKP-1b*	0.241	*18S rRNA*	0.484
6	*Actin*	0.416	*eIF-4a*	0.264	*Actin*	0.505
7	*eEF-1a*	0.586	*Actin*	0.334	*eEF-1a*	0.567
8	*25S rRNA*	0.661	*eEF-1a*	0.353	*25S rRNA*	0.647
9	*UBQ5*	0.685	*UBC*	0.369	*UBC*	0.654
10	*UBQ10*	0.939	*UBQ10*	0.384	*SKP-1b*	0.68
11	*GAPDH*	0.947	*25S rRNA*	0.417	*UBQ10*	1.014
12	*SKP-1b*	0.954	*GAPDH*	0.505	*GAPDH*	1.015
13	*β-TUB*	1.265	*β-TUB*	0.574	*β-TUB*	1.036

Therefore, based on the NormFinder and geNorm analysis, *Fb15*, *17S rRNA* and *eIF-4a* are the best reference genes among the 13 genes in rice endosperm development under normal temperature, while *Fb15*, *UBQ5* and *17S rRNA* are those best for gene normalization in rice endosperm development under high temperature condition.

## Discussion

Real-time PCR is a quick, simple and reliable tool to analyze gene expression. Selection of proper reference genes is one of the major factors that affect the reliability of qRT-PCR. The ideal reference genes should have unchanged expression levels during the whole experimental process and with proper expression intensity. There is no single reference gene that is appropriate for all experimental conditions [7]. Therefore, it is necessary to examine the expression stability of reference genes under specific experimental conditions prior to its use for normalization.

*18S rRNA* was often regarded as the most reliable reference gene for normalization in real-time PCR [20]. However, there are some circumstances when *18S rRNA* cannot be used: (i) reverse transcription reaction is carried out with oligo-dT primer; (ii) mRNA is used as template. Besides, *18S rRNA* is expressed at very high level. When the weakly expressed genes are studied, normalization of gene expression with *18S rRNA* may bias the result. Furthermore, numerous studies have shown that the expression patterns of many housekeeping genes, such as *Actin*, *β-TUB*, *GAPDH*, and *eEF-1a* can vary under certain conditions [22]. Recent studies confirmed that some new candidate reference genes selected based on microarray and transcriptome database showed more stable expression than these traditional reference genes [12, 22]. Our results also confirmed that most of the traditional reference genes such as *Actin*, *β-TUB*, *GAPDH* and *UBQ10* were not as good as *Fb15* when studying gene expression in rice endosperm under high temperature. Therefore, it is particularly efficient and reliable to use high-throughput transcriptome data to identify new reference genes.

Via microarray data analysis, Jain (2009) identified several novel reference genes with relative stable expression at all the 15 developmental stages [4], but qRT-PCR confirmation is required before these reference genes can be used in real research. Li *et al* (2010) also identified *eIF-4a* and *ACT1* as the most stable traditional reference genes during rice seed development (3, 6, 10, 15 and 20 DAP) [23]. These studies did not distinguish the tissues between the embryo and endosperm in rice seeds. However, microarray data from rice embryo and endosperm revealed that gene expression profiles were greatly different in these two tissues [13]. Therefore, these new reference genes identified in rice seed may not be proper for normalization in endosperm development.

On the other hand, endosperm is the major part of rice seed and it determines the crop value both in quantitative and qualitative terms. High temperature affects the rice yield and quality by affecting endosperm development. The transcription profiles undergo enormous changes during endosperm development especially under high temperature [11]. Recently, many studies on the regulatory network of genes related to endosperm development under high temperature have been performed with qRT-PCR approach [24, 25]. However, no research on selection of stable reference genes in rice endosperm development under high temperature has been reported. Here, we selected 6 new candidate reference genes by analyzing DNA microarray and RNA-seq data. None of them was identified as the reference genes previously at various stages of development, including the seeds [4]. Furthermore, we compared the expression variability of two new candidate reference genes with 11 traditional reference genes in endosperm under high temperature treatment by using qRT-PCR analysis. Our results showed that *Fb15*, *UBQ5* and *17S rRNA* have the lowest average expression stability value (M) which means their expression levels were most stable in endosperm under high temperature. It is the first report that *Fb15* can be used as a reference gene. Traditional reference gene *β-TUB* exhibited the most unstable expression among the 11 housekeeping genes, which is consistent with the previous report [4]. Other commonly used reference genes such as *Actin*, *GAPDH*, and *UBQ10* displayed relatively high expression variability which limiting their use as reference genes in research on rice endosperm under high-temperature. The output on gene ranking from NormFinder was slightly different with geNorm, which could be due to the different statistical algorithms applied. Based on the data from NormFinder and geNorm analysis, we conclude that *Fb15*, *17S rRNA* and *eIF-4a* are the best reference genes in rice endosperm under normal temperature, while *Fb15*, *UBQ5* and *17S rRNA* are the genes suitable for expression normalization in rice endosperm under high temperature.

Although *17S rRNA* was identified as the most stable gene in endosperm under high temperature in our study, it cannot be used as a best reference gene in the future due to the same limitations with *18S rRNA* mentioned before. The Ct value of *17S rRNA* in qRT-PCR assay is about 7 to 10, while that of *Fb15*, *eIF-4a* and *UBQ5* are about 19 to 25. Therefore, *Fb15*, *eIF-4a* and *UBQ5* showed proper expression intensity and stability which make them good reference genes. Using the geNorm approach, we also determined that the optimal minimal number of reference genes for normalization gene expression in endosperm under high temperature and normal temperature are both two. Therefore *Fb15* and *UBQ5* are the best reference genes for gene expression studies in rice endosperm under high temperature, while *Fb15* and *eIF-4a* are the most stable genes during rice endosperm development under normal temperature.

## Supporting Information

S1 Table Top 37 genes with most stable expression during rice endosperm development under high temperature. (PDF)
