# Supplementary material for: Genome-Wide Identification of New Reference Genes for qRT-PCR Normalization under High Temperature Stress in Rice Endosperm
Source: PLoS One. 2015 Nov 10;10(11):e0142015. doi: 10.1371/journal.pone.0142015 (PMC4640718; doi:10.1371/journal.pone.0142015)
Supplement: S1 Table — (PDF) [file pone.0142015.s001.pdf]

Table S1. Top 37 genes with most stable expression in rice endosperm development under high temperature.

Gene ID	Mean	SD	CV(%)=SD/Mean	Gene ID	Mean	SD	CV(%)	Functions
		GSE27856				RNA-seq		
LOC_Os01g24690	15434	2229.3	0.14	LOC_Os01g24690.1	56.68	9.65	0.17	60S ribosomal protein L23A, putative, expressed
LOC_Os01g24690	15434	2229.3	0.14	LOC_Os01g24690.2	45.01	7.29	0.16	60S ribosomal protein L23A, putative, expressed
LOC_Os01g38660	277.3	40.6	0.15	LOC_Os01g38660.1	3.26	0.44	0.14	expressed protein
LOC_Os01g48810	1921.7	280.2	0.15	LOC_Os01g48810.1	13.6	1.8	0.13	transcription initiation factor TFIID subunit 11, putative, expressed
LOC_Os01g49026	59.9	5	0.08	LOC_Os01g49026.1	8.73	1.61	0.18	transmembrane protein 93, putative, expressed
LOC_Os01g60530	242.5	36.6	0.15	LOC_Os01g60530.1	2.36	0.41	0.17	transposon protein, putative, CACTA, En/Spm sub-class
LOC_Os01g63890	14090.1	2497.2	0.18	LOC_Os01g63890.1	18.39	3.3	0.18	G10 protein, putative, expressed
LOC_Os01g69980	934.1	131.3	0.14	LOC_Os01g69980.1	1.92	0.37	0.19	TCP family transcription factor, putative, expressed
LOC_Os01g72960	3469.1	427.9	0.12	LOC_Os01g72960.1	7.37	0.55	0.07	expressed protein
LOC_Os02g02890	35411.4	6630.9	0.19	LOC_Os02g02890.1	348.74	66.35	0.19	peptidyl-prolyl cis-trans isomerase, putative, expressed
LOC_Os02g02960	7286.4	878.6	0.12	LOC_Os02g02960.1	3.77	0.39	0.1	histone deacetylase complex subunit SAP18, putative, expressed
LOC_Os02g07910	15329.8	1258.9	0.08	LOC_Os02g07910.1	19	3.26	0.17	fiber protein Fb15, putative, expressed
LOC_Os02g07910	15329.8	1258.9	0.08	LOC_Os02g07910.2	23.21	2.41	0.1	fiber protein Fb15, putative, expressed
LOC_Os02g29500	217.3	34.8	0.16	LOC_Os02g29500.1	2.77	0.42	0.15	expressed protein
LOC_Os02g44100	669	69.5	0.1	LOC_Os02g44100.1	2.23	0.28	0.13	expressed protein
LOC_Os02g56020	2902.4	496.5	0.17	LOC_Os02g56020.2	2.71	0.21	0.08	methyltransferase, putative, expressed
LOC_Os03g06480	7694.7	1199.3	0.16	LOC_Os03g06480.1	12.74	2.39	0.19	expressed protein
LOC_Os03g39820	1328.9	206.6	0.16	LOC_Os03g39820.1	12.55	2.48	0.2	expressed protein
LOC_Os04g02890	177.7	27.1	0.15	LOC_Os04g02890.2	2.1	0.29	0.14	expressed protein
LOC_Os04g31910	1211	72.3	0.06	LOC_Os04g31910.1	4.35	0.18	0.04	expressed protein
LOC_Os05g01710	2872.5	465.5	0.16	LOC_Os05g01710.3	5.27	0.37	0.07	transcription initiation factor IIA gamma chain, putative, expressed
LOC_Os05g23610	3282.7	538.5	0.16	LOC_Os05g23610.2	4.58	0.81	0.18	protein phosphatase inhibitor 2 containing protein, expressed

LOC_Os05g37330	24389.7	2813.8	0.12	LOC_Os05g37330.1	82.72	8.1	0.1	60S acidic ribosomal protein, putative, expressed
LOC_Os05g38520	26797.2	2824.7	0.11	LOC_Os05g38520.1	21.61	2.98	0.14	60S ribosomal protein L36-2, putative, expressed
LOC_Os05g42330	5046.1	709.6	0.14	LOC_Os05g42330.2	9.04	1.77	0.2	secretory carrier-associated membrane protein, putative, expressed
LOC_Os07g01990	9996.9	1621.9	0.16	LOC_Os07g01990.1	7.42	0.97	0.13	expressed protein
LOC_Os07g02000	2304.5	279	0.12	LOC_Os07g02000.1	8.01	1	0.13	expressed protein
LOC_Os07g02210	4692.4	633.8	0.14	LOC_Os07g02210.1	8.46	0.57	0.07	eukaryotic translation initiation factor 5A, putative, expressed
LOC_Os07g02210	4692.4	633.8	0.14	LOC_Os07g02210.3	7.66	1.28	0.17	eukaryotic translation initiation factor 5A, putative, expressed
LOC_Os07g08820	1944.4	361.4	0.19	LOC_Os07g08820.1	8.52	1.51	0.18	C-Myc-binding protein, putative, expressed
LOC_Os08g08084	116.8	15.3	0.13	LOC_Os08g08084.1	0.21	0.02	0.11	expressed protein
LOC_Os08g09860	1301.8	142.8	0.11	LOC_Os08g09860.1	3.67	0.36	0.1	hydroxyacid oxidase 1, putative, expressed
LOC_Os08g18110	15531.8	2784.3	0.18	LOC_Os08g18110.3	12.71	1.74	0.14	alpha-soluble NSF attachment protein, putative, expressed
LOC_Os09g07570	807	133.1	0.16	LOC_Os09g07570.1	5.74	1.04	0.18	ferredoxin-thioredoxin reductase catalytic chain, chloroplast precursor, putative, expressed
LOC_Os09g39350	2052.7	387.3	0.19	LOC_Os09g39350.2	4.95	0.91	0.18	expressed protein
LOC_Os10g33855	4919.3	617.2	0.13	LOC_Os10g33855.2	7.98	1.06	0.13	expressed protein
LOC_Os10g34614	571.2	95.5	0.17	LOC_Os10g34614.4	0.76	0.13	0.17	csAtPR5, putative, expressed
LOC_Os11g02090	159.5	27.1	0.17	LOC_Os11g02090.1	2.15	0.41	0.19	expressed protein
LOC_Os11g26910	12138.1	2286.2	0.19	LOC_Os11g26910.1	71.73	10.59	0.15	SKP1-like protein 1B, putative, expressed
LOC_Os12g04880	592.5	68.4	0.12	LOC_Os12g04880.2	3.79	0.59	0.16	retrotransposon protein, putative, unclassified, expressed
LOC_Os03g50885	7606.3	1070.9	0.14	LOC_Os03g50885.1	138.0	134.1	0.97	actin, putative, expressed
